# The Romanian need of postgraduate specialization in dentistry considering the integration to the European Union

**Published:** 2008-02-25

**Authors:** Stanciu Dragos, Bucur Alexandru, Temelcea Anca, Stanciu Radu

**Affiliations:** * ”Carol Davila” University of Medicine and Pharmacy – Faculty of Dentistry , Bucharest, Romania

## Abstract

Once that Romania became part of the European Union, conditions regarding the activity in dentistry changed, and open borders are now a fact that we have to accept.

The Romanian activity in dentistry developed a lot after the 1990, being a result of private dental practice – which covers over 98% and also of material resources of patients that were asking for more performing dental treatments. In order to be able to face all this, the dentists followed many of postgraduate specializations.

We have to consider as well that the situation of the Romanian dentists is not the best, because they had to face the dentists that got their certificates in Eastern countries, where they only had to pay a fee and not pass an exam. Also, the Romanians that wanted to specialize abroad were not able to do that because the National Dentists’ Association was protecting their own colleagues.

The Romanian Dentists’ Association is obligated to recognize diplomas received in the E.U., but this is not always available for Romanian diplomas once they go to practice abroad.

And this is not enough, because young dentists have limited places if they choose to specialize – 40 places/year for 1500 young dentists. But the possibility to specialize is in areas where there are enough specialists already: Orthodontics – 80%, Dental Surgery – 17%, Oral-Maxillo-Facial Surgery – 1-2%.

What we have to consider is the idea of creating new postgraduate specializations that will cover the need for the Romanian population although this won’t be correlated with the E.U. curricula. Regarding recognition for the Romanian dentist in the E.U., he will be considered a general dentist.

From another point of view, we have to ask the academic environment to help form residents so we will be able to increase the number of the residents/year.

For the Romanian Dentists’ Association, the existence of these postgraduate courses, with the agreement of the Public Health Ministry & the Performance Centre for the Medical System, will be an objective opportunity to recognize foreign diplomas, considering followed curricula. This will stop accepting some certificates and diplomas that are not facing the reality, considering that for the moment, there are no objective criteria to compare them with.

After the visit received from an expert team sent by the E.U. in July 2005, regarding the finalization of medical specialties and curricula – Twinning Light Project RO 2002/IB/OT 03 TL “The harmonization in numbers and content for the medical specialties and curricula, considering the integration in the E.U.”, the Public Health Ministry received a report mentioning that a postgraduate course can appear if needed.

The ideas mentioned in this report were presented and abrogated by the Romanian Dentists’ Association and the Dean’s from all the Romanian Dental Schools.

We present to you the most important arguments that form the basis in comparison with all the other countries in the E.U.:

1. Romanian dental pathology is the richest in the E.U. – DMFT Index is 7.3, 8 times bigger then the one from UK/Switzerland and double in comparison with the one from Albania, which is not even an E.U. country. These dates were presented in the last report made by WHO.

2. Education received by the dental students is formed by general information, meaning that they are not ready to face complicated cases. At the moment, the oral & maxillo-facial cancer and dental anomalies are diagnosed late and the lack in specialists transforms all these treatments in expensive ones and also compromised solutions.

3. Continuous dental education in short periods of time might be a solution, but only for the moment because the need for specialists can not be covered this way.

4. The increasing number in young dentists did not improve the dental health of population or raise medical services’ quality, which justifies the need of postgraduate courses, following the E.U. model.

5. There are some European countries were more medical – dental specialties are accepted, fact accepted also by the European Commission as followed:

- Sweden: periodontology, endodontics, prosthodontics, dento–maxillo facial radiology, dental pathology.

- Germany: periodontology and public health

- Great Britain: public health, pediatric dentistry and operative dentistry

As we can see, each country has the option of choosing the postgraduate courses need by the population they have to help.

In this way, we would like to give as example countries like Finland, Germany, England that have between 3-6 postgraduate courses and the DMFT Index is 6-8 times lower than the Romanian one.

6. In Norway, Poland, Sweden, UK the number of specialists in different dental areas is increased, and this involves an improvement of the public dental health for population.

7. The possibility for patients to choose the dentist should be made considering medical criteria and not commercial aspects.

8. The measures taken in the public health and preventive dentistry has a very small interest shown by the dentists, that makes the efficiency for the dental treatments to decrease and the costs to increase.

Our proposal is to have in dentistry the following specializations:

- Orthodontics – 3 year plan

- Oral and Maxillo-Facial Surgery – 5 year plan

- Periodontology, Implantology and Oral Rehabilitation – 3 year plan

- Preventive Dentistry and Public Health – 3 year plan

- General Dentistry – 3 year plan

We mention that the curricula for each specialization is made according to national competences and are following the E.U. guidelines, that will allow open borders and mutual recognition.

This project will be possible, modifying the organizing law for medical activity by:

- legalizing the specializations mentioned 

Or

- establishing that the number of specialists is to be made by the minister.

